# Diabetes in urban Guinea-Bissau; patient characteristics, mortality and prevalence of undiagnosed dysglycemia

**DOI:** 10.1080/16549716.2020.1802136

**Published:** 2020-08-20

**Authors:** Stine Byberg, Camilla Bundesen, Frauke Rudolf, Thorny Linda Haraldsdottir, Lamine Indjai, Rui Barai, Henning Beck-Nielsen, Morten Sodemann, Dorte Møller Jensen, Morten Bjerregaard-Andersen

**Affiliations:** a Bandim Health Project, INDEPTH Network, Bissau, Guinea-Bissau; b Research Center for Vitamins and Vaccines (CVIVA), Statens Serum Institute, Copenhagen, Denmark; c Department of Clinical Epidemiology, Steno Diabetes Center Copenhagen, Gentofte, Denmark; d Department of Infectious Diseases, Odense University Hospital, Odense, Denmark; e Department of Infectious Diseases, Aarhus University Hospital Skejby, Aarhus, Denmark; f The Diabetes Clinic, The National Diabetes Association (ANDD), Bissau, Guinea-Bissau; g Steno Diabetes Center Odense, Odense University Hospital, Odense, Denmark; h Department of Gynecology and Obstetrics, Odense University Hospital, Odense, Denmark; i Department of Endocrinology, Hospital of Southwest Denmark, Esbjerg, Denmark

**Keywords:** Type 2 diabetes mellitus, Africa, Guinea-Bissau, community burden, risk factors, mortality

## Abstract

**Background:**

The burden of diabetes mellitus in Sub-Saharan Africa is growing rapidly, and yet the prevalence and patient characteristics are still largely unknown.

**Objectives:**

We analyzed clinical and demographic characteristics of Type 2 diabetes (T2DM) patients attending a diabetes clinic in Guinea-Bissau from February 2008 to April 2014, and estimated the prevalence and risk factors of unknown-impaired fasting plasma glucose (FPG) and diabetes, as well as excess mortality associated with T2DM.

**Methods:**

We characterized T2DM patients attending the national diabetes clinic in Bissau. Diabetes was diagnosed based on FPG. We matched T2DM patients 1:1 with non-diabetes community controls on age and sex to determine relevant risk factors for T2DM using logistic regression. Furthermore, we matched patients 1:6 with community controls to assess long-term survival (until February 2019) in a Cox regression using calendar time as the underlying timescale. Verbal autopsies determined the cause of death among T2DM patients and controls.

**Results:**

The mean age among T2DM was 50.6 (SD 11.1), and the mean FPG at first consultation was high (13.2 mmol/L (SD 5.1)). Ethnicity, family history of diabetes, hypertension, and anthropometrics differed among T2DM patients, community controls with impaired FPG, and healthy controls. Family history of diabetes (OR = 5.65, 95% CI: 3.10–10.3) and elevated waist circumference (2.33, 1.26–4.29) were significant risk factors for T2DM. 20.4% (59/289) of community controls had abnormal FPG. T2DM patients had an excess mortality hazard ratio of 3.53 (95%CI: 1.92–6.52). Deaths caused by bacterial infections, including foot ulcers, were more common among T2DM patients, compared with community controls (54% (7/13) vs. 19% (5/27) (P = 0.02)).

**Conclusion:**

Several risk factors including were associated with T2DM in Guinea-Bissau. We found a high prevalence of elevated FPG among randomly selected community controls. In combination with higher mortality among T2DM patients, an urgent need for better treatment options and increased awareness.

## Background

The prevalence of diabetes mellitus in Sub-Saharan Africa has increased rapidly over the past decades and is expected to more than double by 2040 [1,2]. The overall diabetes prevalence in Africa is estimated to be 3.9% [3], though substantial inter- and intra-country differences exist [2]. However, epidemiological diabetes data from Africa remain scarce, with many countries in sub-Saharan Africa lacking valid data [3]. Very few studies have been conducted in West Africa; in a study from Dakar, Senegal, the prevalence of diabetes was 17.9% [4]. In contrast, the prevalence of diabetes in rural Senegal was much lower, i.e. 4.2% [5], suggesting differences in background risk factors and exposures. In a clinic-based study in Bo, Sierra Leone, the overall diabetes prevalence was 6.2% [6], while it was 5.8% in a sample from Burkina Faso [7].

Up to 70% of diabetes patients in Sub-Saharan Africa, however, very likely remain undiagnosed and therefore do not receive treatment [3]. Thus, in a study from Mauritania, two-thirds of the identified diabetes patients were unaware of their condition [8]. These untreated individuals have a high mortality and many diabetes-related complications.

At the Health and Demographic Surveillance System (HDSS) site maintained by the Bandim Health Project (BHP) in Guinea-Bissau, we have previously assessed the prevalence of diabetes among specific groups such as HIV patients [9], tuberculosis patients [10] and twins [11,12]. The diabetes prevalence among adult controls in the tuberculosis study was 2.7%, while it was 5.8% among HIV patients. A broader epidemiological study focusing on diabetes patients in the general population, including mortality, has however not been conducted.

We determined the number of Type 2 diabetes (T2DM) patients attending the only diabetes clinic in Guinea-Bissau from February 2008 to April 2014, including clinical and demographic characteristics, treatment, and risk factors. Furthermore, we estimated the prevalence and risk factors of unknown-impaired fasting glucose (IFG) and diabetes in the general population. Finally, we calculated the excess mortality associated with T2DM, and assessed differences in causes of death among T2DM patients and community controls.

## Methods

### Setting

The study was conducted in the six suburban areas under surveillance by the BHP in the capital Bissau, Guinea-Bissau. The suburbs have a population of approximately 103,000 individuals. Censuses are conducted regularly. All individuals are assigned a unique ID-number. Date of birth, sex, ethnic group, and socio-economic characteristics are registered. Pregnancies and births, as well as deaths and migration in or out of the area, are recorded.

### Diabetes clinic

The only diabetes clinic in Guinea-Bissau is situated within the BHP study area. The clinic is semi-private and run by the Guinean Diabetes Association (ANDD). Patients are referred from other health facilities or self-refer. No uniform referral process exists. The clinic registers all first-time patients and completes a clinical record, including demographic characteristics, patient history, anthropometry, clinical measurements, prescriptions, diagnoses, and the presence of foot ulcers. These questionnaires have been altered over the years; the questionnaires from the beginning of the period had less information. The questionnaires are completed in the local language Portuguese Creole.

We included all T2DM patients diagnosed at the clinic between February 2008 and April 2014. For most of the study period, the clinic was staffed by a local doctor and a nurse, with assistants providing auxiliary functions. The clinic was mainly financed by patient fees, with first-time patients paying 2500 CFA (~5 USD) for a consultation. For control visits, the fee was 500 CFA (~1 USD). Fees were sometimes waived in case severely ill patients could not pay. Patients with T2DM were normally prescribed anti-diabetic drugs and other medicines, which they paid out-of-pocket at local pharmacies. Insulin was expensive and often not available at all.

When diagnosed with diabetes, patients entered a routine schedule, where weight, blood pressure, and fasting plasma glucose (FPG) were measured and treatment adjusted accordingly. The frequency of these visits varied.

### Measurements at the diabetes clinic

At the clinic, height, hip-, waist- and middle upper-arm circumference (MUAC) were measured with a standard measuring tape. Weight was measured using a standard bathroom scale.

Blood pressure and pulse were measured manually until February 2014 when an automatic device became available.

All first-time patients underwent a blood glucose test with an Accu-Chek Aviva apparatus which displayed results as capillary plasma glucose, using finger prick blood. The test was usually done after an overnight fast.

Both Accu-Check Aviva apparatuses, and the anthropometric measurements, have previously been applied by the BHP [10].

### Diabetes definitions

In accordance with the World Health Organization, we defined diabetes as FPG ≥7.0 mmol/L, or a random blood glucose of ≥11.1 mmol/L [13]. FPG between 6.1 and 6.9 mmol/L was considered impaired fasting glucose (IFG) [13]. Due to the limited clinical parameters available, the distinction between T1DM and T2DM was done based on a clinical assessment, including age and BMI. In this report, we focus on T2DM.

### Patients included

We reviewed all medical records of patients treated at the diabetes clinic (patients aged 27 to 89 years). All T2DM patients reporting to be living within the BHP study area were included in the risk factor analyses. Only T2DM patients who we could identify with certainty through the HDSS database were included in the mortality analysis, as the follow-up required a valid HDSS ID number.

### Risk factors for T2DM

We assessed the distribution of risk factors in T2DM patients at the clinic and in 1:1 age- and sex-matched community controls (non-diabetes) from the BHP study area. Community controls were selected randomly from the HDSS database and visited at home the day before the planned glucose measurement. A trained field assistant explained about the study and informed consent was obtained. The community controls were instructed to fast overnight and visited at the household early in the morning and FPG was measured. If FPG was found to be elevated, indicating IFG or diabetes, a new age and sex-matched control were identified. Those identified with high FPG were referred to the clinic. All controls completed the same questionnaire as the T2DM patients.

We included risk factors based on the available literature, particularly with reference to African studies [1,14–16]. We examined several potential risk factors; ethnicity, family history of diabetes, family history of hypertension, any alcohol intake, any tobacco smoking, BMI, and elevated waist circumference (86 cm for men and 92 cm for women, in accordance with African recommendations [17]).

### Mortality

We randomly selected age- and sex-matched community controls 6:1 from the BHP HDSS database. Hence, for a given T2DM patient from the BHP area, six random age- and sex-matched controls, alive at the day of the initial diabetes consultation of the T2DM patient, were selected and followed. Only T2DM patients with a valid BHP ID were included, as this was a prerequisite for follow-up. Both T2DM patients and community controls were followed by the HDSS database until February 2019.

We performed verbal autopsies (VA) among all deaths registered among the T2DM patients and community controls. VA interviews of close family members were conducted by a trained fieldworker and afterward reviewed by a BHP physician. We used a slightly modified version of a VA questionnaire previously applied to tuberculosis patients at our site [18].

## Statistics

We assessed the distribution of background factors among T2DM patients and community controls (matched 1:1), using the chi-square test for categorical variables and ANOVA for continuous variables. We divided community controls into controls with normoglycemia and controls with IFG/diabetes. Subsequently, we calculated odds ratios (OR) of selected risk factors for having T2DM with 1) community controls with normoglycemia and 2) community controls with IFG/diabetes, respectively. We calculated the ORs for risk factors in both univariate and multivariate models, using logistic regression. We included ethnicity, gender, family history of diabetes, and hypertension, as well as alcohol and tobacco use, and anthropometrics previously reported as risk factors for T2DM in Africa [14].

We compared the hazard rates of death among T2DM patients vs. community controls (matched 1:6) in a Cox regression model, using time in years since cases’ first consultation at the diabetes clinic as the underlying timescale. Adjustment was made for the matching variables age and gender.

All statistical analyses were done in STATA software (Stata Corporation, College Station, TX, USA).

## Results

### T2DM patients at the clinic

#### Number of patients

**Figure 1.**
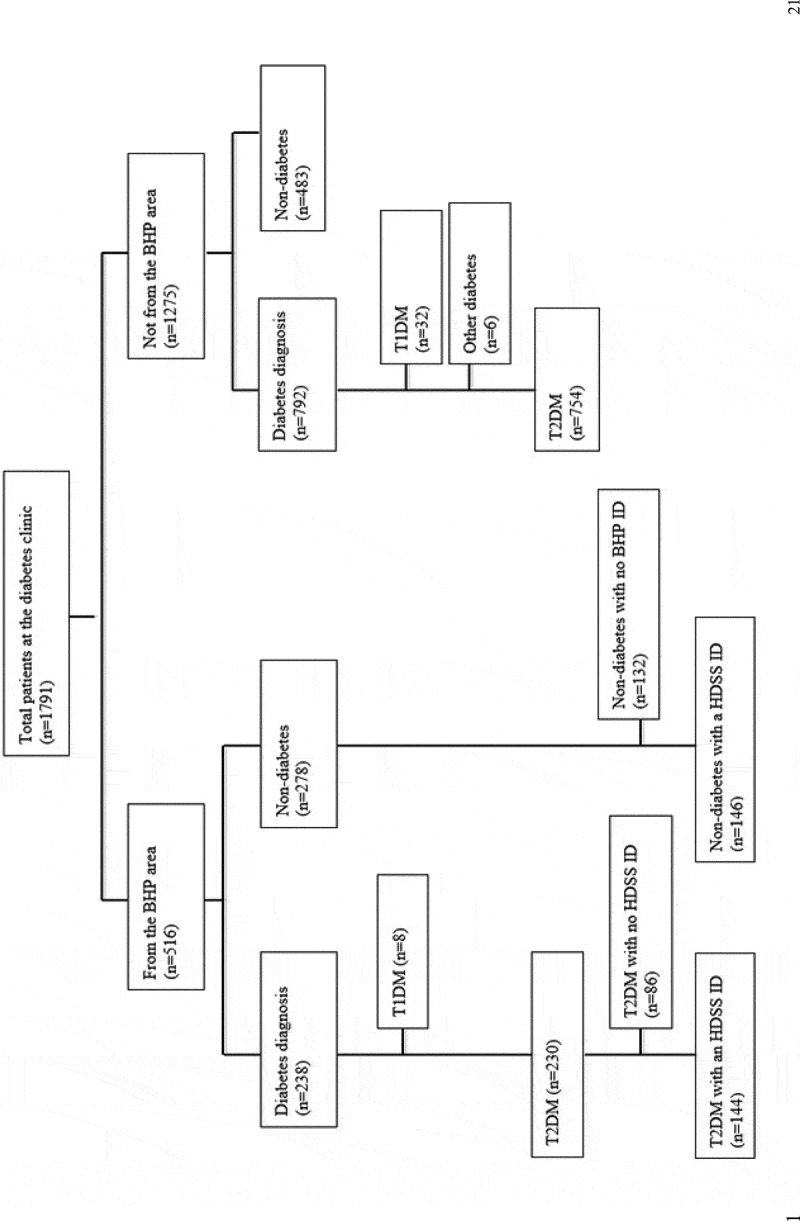
Study flowchart 2008–2014.

From February 2008 to April 2014, 1791 persons attended the diabetes clinic at least once; 516 living within the BHP study area and 1275 from other parts of Bissau city or the rural interior of the country. After reviewing the records, 1030 patients either had known diabetes or diabetes was diagnosed at the clinic. Of these, a total of 984 patients were classified as T2DM, with 230 reporting to live within the BHP study area (see Figure 1).

Among T2DM patients reported to be living in the BHP study area, the number of new patients per year increased from 20 new patients in 2009 to 51 in 2013 . The biggest increase was seen in 2011. We were subsequently able to identify 62.6% (144/230) of the patients in the HDSS database.

#### Clinical characteristics

Among T2DM patients reporting to be living within the BHP study area, 53% (121/230) were female (Table 1). The mean age was 50.6 years (SD 11.1 years). Mean BMI was 26.3 kg/m^2^ overall, though higher for women than men, 27.9 kg/m^2^ vs. 24.6 kg/m^2^, respectively. A total of 9% (19/212) had a BMI above 35 kg/m^2^ (severe obesity). The mean waist circumference was 87.6 cm for males and 91.0 cm for females. Fifty-six percent (40/72) of males and 48% (40/83) of females had an elevated waist circumference. Twenty-five percent (56/226) had elevated systolic blood pressure (>140 mmHg).

**Table 1.** Demographic characteristics, patient history, anthropometry, and clinical measurements at inclusion for T2DM patients, healthy community controls, and community controls identified with IFG/diabetes, from the BHP study area.

	T2DM	Community controls without IFG	Community controls with IFG/diabetes	P-value
Demographics				
Sex				
- Male	47.4% (109/230)	45.9% (105/229)	52.5% (31/59)	0.64
- Female	52.6% (121/230)	54.4% (125/230)	47.5% (28/59)	
Age; years (mean (SD))	50.6 (11.1)	50.4 (11)	53.1 (9)	0.23
- < 35 years	7.9% (18/229)	7.8% (18/230)	0 (0/59)	
- 35 – 45 years	23.1% (53/229)	23.5% (54/230)	18.6% (11/59)	
- 45 – 55 years	34.9% (80/229)	35.7% (82/230)	45.8% (27/59)	
- 55 – 70 years	29.3% (67/229)	28.3% (65/230)	32.2% (19/59)	
- >70 years	4.8% (11/229)	4.8% (11/230)	3.4% (2/59)	
Ethnicity				
- Pepel	17.8% (41/230)	28.3% (65/230)	11.9% (7/59)	<0.001
-	7.8% (18/230)	8.3% (19/230)	5.1% (3/59)	
- Balanta	16.5%(38/230)	20.0% (46/230)	18.6% (11/59)	
-Mandinga/Fula	28.7% (66/230)	13.4% (30/230)	27.1% (16/59)	
- Mancanha/Manjaco	18.3% (42/230)	41.3% (95/230)	37.3% (22/59)	
- Other				
Patient history				
History of co-morbidity				
- Typhoid fever	31.8% (47/148)	16.7% (38/227)	25.9% (15/58)	0.003
- Hepatitis	9.7% (14/145)	12.8% (29/227)	17.2% (10/58)	0.32
- Tuberculosis	4.4% (7/159)	4.8% (11/230)	3.5% (2/57)	0.92
- HIV/AIDS	1.0% (1/92)	2.2% (5/228)	0% (0/59)	0.44
- Hypertension	55.8% (86/154)	42.3% (82/191)	65.3% (35/49)	0.005
- Malaria	23.9% (55/230)	95.2% (216/227)	5.1% (3/59)	<0.001
Family history				
- Diabetes	55.0% (82/149)	12.6%% (20/159)	39.0% (16/41)	<0.001
- Hypertension	57.3% (79/138)	36.7% (62/169)	63.0% (29/46)	<0.001
Alcohol intake (any)	57.8% (123/213)	54.8% (126/230)	54.2% (32/59)	0.79
Amount of alcoholic beverages				
- 1/month	17% (13/77)	12.6% (16/127)	17.2% (5/29)	0.07
- 1-3/week	56% (43/77)	64.6% (82/127)	72.4% (21/29)	
- 2-3/week	5% (4/77)	0% (0/127)	0% (0/29)	
- every day	22% (17/77)	22.8% (29/127)	10.3% (3/29)	
Tobacco smoking				
Amount of smoking	11.1% (23/208)	12.6% (29/230)	8.5% (5/59)	0.65
- 1 cigarette/month	0% (0/18)	0% (0/26)	0% (0/7)	0.68
- 1-5 cigarettes/week	17% (3/18)	19% (5/26)	42.9% (3/7)	
- 6-10	28% (5/18)	31% (8/26)	28.6% (2/7)	
cigarettes/week				
>10 cigarettes/week	39% (7/18)	27% (7/26)	28.6% (2/7)	
- Pipe	17% (3/18)	23% (6/26)	0% (0/7)	
Anthropometry				
Weight; kg	72.5 (15)	69.9 (17)	77.5 (15)	0.004
Height; cm	166 (8)	167 (9)	167 (8)	0.74
BMI; kg/m^2^	26.3 (5)	25.2 (6)	27.7 (5)	0.005
- Male	24.6 (4)	23.8 (4)	27.7 (4)	<0.001
- Female	27.9 (6)	26.3 (7)	27.7 (6)	0.15
BMI categories				
- <25	44.8% (95/212)	56.3% (129/229)	25.4% (15/59)	0.001
- 25-35	46.2% (98/212)	38.9% (89/229)	66.1% (39/59)	
- >35	9.0% (19/212)	4.8% (11/229)	8.5% (5/59)	
Upper arm circumference; cm	293 (35)	292 (49)	311 (39)	0.01
- >320 mm	21.7% (25/115)	22.6% (52/230)	37.2% (22/59)	0.05
Waist circumference; cm				
- Male	90 (13)	85 (12)	94 (12)	<0.001
- Female	88 (12)	85 (11)	97 (10)	<0.001
Elevated waist circumference	91 (14)	85 (13)	90 (13)	0.005
-Male (>86cm)	55.6% (40/72)	38.1% (40/105)	83.9% (26/31)	<0.001
-Female (>92 cm)	48.2% (40/83)	24.0% (30/125)	46.4% (13/28)	0.001
Clinical				
FPG level at initial cons.: mmol/L	13.2 (5.1)	5.2 (0.4)	8.1 (3.3)	<0.001
- 0-6.9	8.2% (11/135)	100% (230/230)	54.2% (32/59)	
- 7.0-11.1	32.6% (44/135)	0/230	30.5% (18/59)	
-11.2-15	20.0% (27/135)	0/230	11.9% (7/59)	
->15.1	39.3% (53/135)	0/230	3.4% (2/59)	
Blood pressure; mmHg				
- Systolic	130 (28)	147 (30)	160 (31)	<0.001
- Diastolic	80 (14)	89 (14)	92 (13)	<0.001
- Systolic>140 mmHg	24.8% (56/226)	51.1% (117/229)	69.5% (41/59)	<0.001
- Diastolic>90 mmHg	15.9% (36/226)	45.9% (105/229)	49.2% (29/59)	<0.001

Cells are % (n/N) or mean (SD) unless otherwise noted.

In total, 39% (82/209) reported a family history of diabetes, while 40% (79/199) reported a family history of hypertension. Eleven percent (23/208) were smokers, while 22% (17/77) had a daily alcohol intake. Only 0.7% (1/154) reported being HIV infected, while 4.4% (7/159) reported previous or concurrent tuberculosis.

The mean FPG among fasting patients was 13.2 mmol/L upon presentation, with 39% (53/135) having an FPG above 15.1 mmol/L. Upon examination, 12% (26/211) had foot ulcers. Characteristics of community controls can be seen in Table 1.

#### Treatment

In terms of anti-diabetic treatment, 64% (147/230) were either prescribed or already in metformin treatment at the time of the first consultation, while 65% (149/230) received sulfonylureas (most often glibenclamide). In total, 60% (131/230) received a combination of the two. A combination drug (metformin/glibenclamide) was used in 3% (6/230) of the patients. Only 2% (5/230) received insulin.

For anti-hypertensive treatment, 16% (37/230) were in ACE-inhibitor treatment, usually captopril. Seven percent (16/230) received a thiazide drug. We did not have data on lipid-lowering treatment, but many patients were prescribed vitamin supplementation.

#### Glycaemic regulation during follow-up

Of the 230 T2DM patients from the BHP study area, 167 attended at least one subsequent control visit at the clinic. Of these, 96 reported being fasting at the initial and second visits. Among fasting patients, 75% (72/96) showed improved glycaemic control at the second visit. Only 20% (19/96) had, however, an FPG below 7.0 mmol/L at the second visit. The average time between the two visits was approximately 2 months (63 days).

### Risk factor analyses

During 2014, 289 randomly selected age- and sex-matched controls from the BHP study area were visited at their household. A total of 20% (59/289) had an abnormal fasting glucose level and were therefore censored in the initial risk factor analysis (12% (34/289) had IFG, while 9% (25/289) were above the diabetes threshold).

We compared background factors among T2DM patients attending the diabetes clinic and matched community controls with normal FPG (Table 2), and compared with community controls with IFG/diabetes (Supplementary Table 1). Since complete information was missing for some individuals, the multivariate model only comprised 373 individuals. Family history of diabetes (OR = 5.65, 95% CI: 3.10–10.3) and elevated waist circumference (2.33, 1.26–4.29) were significant risk factors for T2DM, compared with community controls with normoglycemia (Table 2). Likewise, Mandinga/Fula ethnicity had higher odds of T2DM, compared with Pepel ethnicity.

**Table 2.** Risk factors for T2DM, compared with community controls without IFG/diabetes (i.e. normoglycemic controls).

	Univariate (OR)	Multivariate (OR) N = 373
Ethnicity		
Pepel	1.00 (ref)	1.00 (ref)
Balanta	1.50 (0.73–3.09))	2.15 (0.92–5.01)
Mandinga/Fula	2.03 (1.17–3.54)	2.44 (1.12–5.34)
Mancanha/Manjaco	0.94 (0.56–1.60)	0.70 (0.35–1.39)
Others	3.77 (2.26–6.30)	1.56 (0.78–3.14)
Gender^a^		
Female	1.00 (ref)	1.00 (ref)
Male	1.06 (0.74–1.54)	1.23 (0.73–2.09)
Patient history		
Family history of diabetes^b^	6.74 (3.95–11.5)	5.65 (3.10–10.3)
Family history of hypertension^c^	1.78 (1.19–2.68)	1.30 (0.77–2.19)
Alcohol intake (any)^d^	1.13 (0.77–1.64)	1.27 (0.72–2.23)
Tobacco smoking (any)^e^	0.86 (0.48–1.54)	1.03 (0.48–2.19)
Anthropometry		
BMI^f^		
<25	1.00 (ref)	1.00 (ref)
25–35	1.50 (1.01–2.21)	0.89 (0.50–1.60)
>35	2.35 (1.07–5.16)	1.35 (0.45–4.03)
Elevated waist circumference^g^	2.47 (1.62–3.78)	2.33 (1.26–4.29)

^a^1 missing

^b^22 missing

^c^31 missing

^d^17 missing

^e^22 missing

^f^18 missing

^g^Above 86 cm for men, 92 cm for women; 76 missing

BMI was in general higher among community controls with screen-detected IFG/diabetes, compared with known T2DM patients attending the diabetes clinic, although not significantly (Supplementary Table 1). Due to relatively few individuals with complete information in this analysis (n = 123), we mainly saw tendencies towards increased risk of screen-detected IFG/diabetes with elevated waist circumference, a family history of hypertension and Mancanha/Manjaco, Mandinga/Fula and other ethnicities compared with Pepel ethnicity. Community controls with IFG/diabetes tended to have lower odds of family history of diabetes, compared with persons with known T2DM.

Among T2DM patients, 144 had a HDSS ID available for follow-up. Of these, 36 had last been seen and followed up by the HDSS team before their first consultation at the diabetes clinic and were therefore excluded. In total, 108 T2DM patients and 648 random age- and sex-matched community controls were therefore included in the survival analysis (1:6 match).

**Figure 2.**
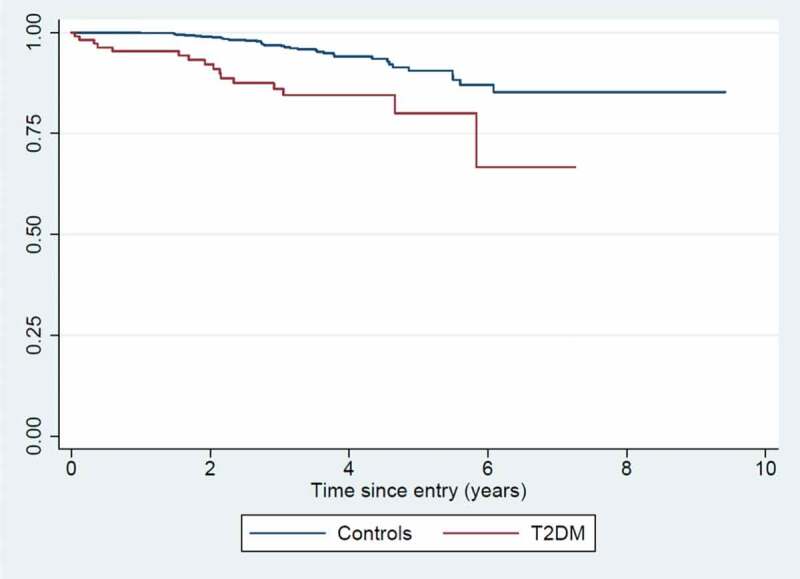
Kaplan-Meier survival curve for T2DM patients vs. community controls.

The average follow-up time was 3.5 years, with follow-up continuing until February 2019. For T2DM patients, the mortality rate (MR) was 46.9/1000 person-years (16 deaths in total). For community controls, the MR was 15.2/1000 person-years (34 deaths in total). The mortality rate ratio (MRR) was 3.53 (CI: 1.92–6.52), adjusted for age and sex (Figure 2).

#### Verbal autopsies

Out of the 16 deaths among T2DM patients, a VA was obtained for 13 individuals. For the remaining three, no family members could be identified. Among the 13 with VA, diabetes was confirmed among relatives in all cases. The primary causes of death were: seven bacterial infections (including foot ulcers), one HIV infection, three cardiovascular/cerebrovascular incidents, one diabetes-related metabolic complication, one liver failure.

For the 34 deaths among community controls, VA was obtained for 27 individuals. According to the VA interviews, three of the deceased controls in the survival analysis had diabetes. The primary cause of death was: five bacterial infections, three tuberculosis infections, 2 HIV infections, eight cardiovascular/cerebrovascular events, one accident, one case of asthma, one liver failure, one renal failure, one snake bite, one case of cancer.

Death from bacterial infection, including infected foot ulcers, was more common among T2DM patients, 54% (7/13) vs. 19% (5/27) (P = 0.02).

## Discussion

### Main findings

We identified an increasing number of T2DM patients at the diabetes clinic. Approximately 39% presented with FPG levels above 15.1 mmol/L, indicative of severe glycemic dysregulation. Family history of diabetes and elevated waist circumference was significant risk factors. Approximately 20% of the randomly selected community controls had an abnormal fasting glucose level (either IFG or diabetes), indicating a substantial community burden. T2DM patients had a 3.5 fold excess mortality, compared to age- and sex-matched controls from the background population.

### Consistency with previous findings

The T2DM burden is currently increasing in Sub-Saharan Africa [1,15]. This may be reflected in our findings of increased attendance to the diabetes clinic over the study period. Yet, a more likely explanation is that diabetes awareness in the population increased. Due to a large grant from the World Diabetes Foundation, awareness activities had been conducted through radio programs and campaigns, and also at health centers in Bissau. This probably resulted in more people being tested. The activities were launched in the second half of 2011, which coincided with the increase in patients. However, assuming that all patients with diagnosed diabetes in Guinea-Bissau attended the diabetes clinic, the diabetes prevalence in the present study is far lower than the estimates from the International Diabetes Federation (IDF) of 186,000 persons with diabetes in Guinea-Bissau [3].

Only a few other diabetes studies have been conducted in Guinea-Bissau, with the focus on diabetes in specific groups. In a tuberculosis study, we found a T2DM prevalence of 2.1% (11/531) among non-tuberculosis community controls [10], while the prevalence was 5.8% (52/893) in patients at the main HIV clinic in Bissau, prior to the initiation of antiretroviral treatment [9]. One study assessed diabetes in a cohort of 1119 police officers. Using random blood glucose measurements, with HbA_1c_ for confirmation, a diabetes prevalence of 4.1% was found [19]. In all three studies, the participants were younger than in our sample, which may explain why our study found a higher community burden of T2DM, i.e. 9%. A recent study among personnel at four military complexes in Guinea-Bissau found a very high diabetes burden of 14.3% (68/476) using FPG [20]. The median age in this study was 43 years, and notably the study population was almost entirely male (94%). This, combined with the likely affluence of many of the participants, may explain the observed discrepancy between this and the other diabetes studies conducted in Guinea-Bissau. Using HbA1_1c_ as both screening and diagnostic tool, a twin study observed a prevalence of merely 0.2% (1/463) among the singleton control group, but the participants in this study were often teenagers, with a mean age of 15.8 years [11].

Undiagnosed T2DM remains a huge problem in Sub-Saharan Africa. The IDF estimated that 127,000 persons in Guinea-Bissau had undiagnosed diabetes in 2019 [3], equivalent to approximately 7% of the population. In our study, the prevalence of undiagnosed diabetes was slightly higher (9%). In some countries, it has been estimated that up to 75% of all T2DM cases remain unknown [15]. This has a big impact on diabetes-related mortality. Thus, assuming our identified T2DM burden of 9% represents the ‘true’ value in the general population (adult studies ranging from 2.1%-14.3%), a prevalence of this magnitude was not reflected in the number of patients actually seen at the diabetes clinic. This indicates that T2DM is most likely vastly underdiagnosed in our setting.

In Sub-Saharan Africa, T2DM patients are often diagnosed quite late, when severe complications have already begun to manifest [16]. This has also been our experience. In the present data, 39% of T2DM patients presented with FPG levels above 15.1 mmol/L (severely dysregulated). Twelve percent of T2DM patients presented with late manifestations of diabetes complications (foot ulcers). This is in line with findings elsewhere [16].

In terms of treatment, it was striking that only 2% of the patients at the diabetes clinic received insulin. Most likely, this was a direct result of poor insulin availability, combined with high costs and poor cold chain storage possibilities. As only few patients purchase insulin in Guinea-Bissau, the pharmacies are reluctant to purchase more insulin. Often patients are forced to go to neighboring country Senegal to purchase insulin. Metformin, often combined with an older sulfonylurea drug such as glibenclamide, was usually the treatment of choice.

Considering risk factors for T2DM in our setting, elevated waist circumference – as a measure of abdominal fat distribution – was strongly associated with T2DM, a finding also was seen elsewhere in Africa [14,21].

Among non-modifiable factors, particularly the family history of diabetes was highly important and was associated with almost six times higher odds of T2DM, compared with normoglycemia community controls. Some ethnicities also seemed more disadvantaged. Future research could seek to clarify whether this is due to genetic disposition as opposed to cultural habits, e.g. higher intake of fat and oily food. Ethnicity as a risk factor has previously been reported [15]. Thus, in the diabetes study among police officers from Bissau particularly Fulas had an excess risk, which was also confirmed in the present study.

Compared with the background population, we observed an almost fourfold-fold higher mortality rate among T2DM patients, over an average of the 3.5 years follow-up period. In high-income settings, an excess mortality among T2DM patients of 1.5–2.5 has been reported [22]. Unlike high-income countries, T2DM deaths in Africa are often due to acute metabolic complications and infections [15]. A large-scale review based on available studies from Nigeria identified hyperglycemic emergencies, diabetic foot ulcers, and cardiovascular disease as the most prevalent diabetes complications [23]. In our VA data, deaths from bacterial infection, including infected foot ulcers, were significantly more common among the T2DM patients.

Few other longitudinal diabetes studies are available from Sub-Saharan Africa. One recent study from Cameroon, however, followed 628 T2DM outpatients for an average of 3 years [24]. The mortality rate was 25/1000 person-years, i.e. approximately half the rate we observed. In the Cameroonian study, mean HbA_1c_ at entry was 8.6% (corresponding to an average blood sugar level of 11.1 mmol/L), indicating a better glycaemic regulation than in our study. Also, more individuals received insulin in the Cameroonian study, and the rate of foot ulcers was only about one-fourth of what we observed.

### Implications and recommendations

It is evident that T2DM is already an important health issue in Guinea-Bissau. Assuming Guinea-Bissau will experience the same rapidly increasing diabetes rates as predicted elsewhere in Africa, the problem will become only bigger within the coming decades [1,15]. Thus, there is a need for a comprehensive national diabetes policy, which at the moment does not exist. A recent study identified many shortcomings in the diabetes care, including lack of specialists and resources for treatment and care, low awareness, and no standardized protocols for diabetes management [25].

In terms of prevention, diabetes awareness activities and campaigns should be expanded. Additional focus should be placed on the dangers of obesity, including recommendations for physical exercise and healthy diet [26]. Testing should also be particularly recommended in case of family history of diabetes.

The high excess mortality associated with T2DM shows that it is currently not a well-treated disease in Bissau. Lack of diabetes specialists, knowledge on the disease, more sophisticated diabetes management tools such as HbA_1c_ assays are probably part of the explanation, but also that drugs are often out of supply. Furthermore, many patients cannot afford the necessary diabetes drugs, are non-compliant with their medication, or do not turn up regularly for control. In our data, only 20% of T2DM patients had reached acceptable glycemia levels at the second visit to the clinic. Better strategies are needed, including making essential drugs such as insulin, metformin, and other anti-diabetic agents available at a reduced cost. Follow–up of diabetes patients for adequate glucose control also need to be improved.

### Strengths and limitations

The study was conducted at the only diabetes clinic in Guinea-Bissau, thus comprising all diabetes patients receiving treatment and followed for their diabetes in Guinea-Bissau. Furthermore, the data were linked to an elaborate HDSS, facilitating sampling of matched community controls and conducting long-term survival analyses. To our knowledge, this is the only longitudinal diabetes investigation from Guinea-Bissau, and indeed one of the very few from the entire African region. Furthermore, we were able to assess the prevalence of screen-detected IFG and diabetes in a random (albeit small) sample of community controls.

The study, however, has several weaknesses. Most important were the diagnostic limitations in Guinea-Bissau, which meant that diabetes was diagnosed based on fasting blood glucose alone. HbA_1c_ measurement, which has been recommended for diagnosing diabetes [27], was not available. Also, the differentiation between T2DM and T1DM was done based on age and phenotypic presentation, i.e. without any advanced biomarkers such as C-peptide or GAD-antibodies. Some individuals may, therefore, have been wrongly classified, though the study investigators sought to limit this by rigorously reviewing all clinical records. In case of doubt whether an individual had diabetes, the person was classified as not having it.

Many patients were self-referred to the clinic. Hence, the results could be influenced by selection bias, where patients with a higher level of education, higher affluence, urban residency, or family history of diabetes were more likely to attend.

A substantial number of community controls (identified by our field survey) had to be censored from the risk factor analysis due to high FPG levels, i.e. above the IFG/diabetes threshold. Although the community controls were instructed to fast overnight before their FPG measurement and fasting was confirmed on the day of measurement, we cannot be certain that the high prevalence of IFG/diabetes is not a result of non-fasting. Again, the use of an HbA_1c_ assay would have been of great value [27].

Only 63% of the T2DM patients could be identified with an ID in the HDSS. This limited the number of T2DM patients for the survival analysis. The problem with missing IDs was most pronounced for the earlier records.

In the mortality analysis, we cannot rule out that the ‘healthy controls’ had diabetes, as we did not measure their blood glucose levels. Three deceased controls were in fact reported by their relatives to have diabetes. However, if unidentified diabetes patients were among the healthy controls, censoring them would only increase the MRR.

The cause of death for deceased T2DM patients and controls was established by VA. Naturally, this method has a degree of uncertainty, as it is based on family members’ recollection of events. However, the VA has been recognized by the World Health Organization as a valuable tool in low-income countries [28].

## Conclusion

Our study confirms that T2DM is a substantial problem in Guinea-Bissau, with many new cases annually and high rates of undiagnosed IFG and diabetes. Of particular concern was the almost fourfold excess mortality associated with T2DM, even when receiving treatment. This calls for immediate action in terms of improving treatment and diabetes awareness in the country. A pressing problem is the lack of availability of essential drugs, such as insulin, and the out-of-pocket payments for medicines, which may keep several patients from adhering to treatment. It is imperative that a national strategy is put in place both for the prevention and treatment of diabetes and for the availability of essential antidiabetic drugs at low cost. Furthermore, a more comprehensive study of the diabetes prevalence and diabetes complications is needed to truly ascertain the burden of diabetes in Guinea-Bissau.

## Supplementary Material

Supplemental Material

## Paper context

Diabetes is increasing rapidly in Africa. However, it is generally not well diagnosed and managed. Good epidemiological data are essential for developing strategies to prevent and manage diabetes, but such data are generally lacking for Sub-Saharan Africa. In this paper, we assess the risk factors for having diabetes, the increased mortality risk associated with diabetes and the prevalence of impaired fasting glucose and diabetes in a random community sample in Bissau, Guinea-Bissau.
