# Supplementary material for: Diabetes in urban Guinea-Bissau; patient characteristics, mortality and prevalence of undiagnosed dysglycemia
Source: Glob Health Action. 2020 Aug 20;13(1):1802136. doi: 10.1080/16549716.2020.1802136 (PMC7480585; doi:10.1080/16549716.2020.1802136)
Supplement: Supplemental Material [file ZGHA_A_1802136_SM3985.docx]

**Supplementary table 1:** Risk factors for having screening detected IFG/diabetes among community, controls compared with known T2DM patients attending the diabetes clinic.

|  | **Univariate (OR)** | **Multivariate (OR)**  N=123 |
| --- | --- | --- |
| Ethnicity |  |  |
| Pepel | 1.00 (ref) | 1.00 (ref) |
| Balanta | 0.98 (0.21-4.54) | 0.64 (0.05-9.02) |
| Mandinga/Fula | 1.40 (0.50-3.95) | 2.04 (0.30-13.9) |
| Mancanha/Manjaco | 3.12 (1.14-8.54) | 4.96 (0.86-28.6) |
| Others | 1.36 (0.54-3.42) | 8.64 (1.54-48.5) |
| Gender |  |  |
| Female | 1.00 (ref) | 1.00 (ref) |
| Male | 1.23 (0.69-2.18) | 0.63 (0.22-1.77) |
| Patient history |  |  |
| Family history of diabetes^1^ | 0.52 (0.26-1.06) | 0.44 (0.18-1.10) |
| Family history of hypertension^2^ | 1.27 (0.64-2.53) | 1.60 (0.60-4.28) |
| Alcohol intake (any)^3^ | 0.87 (0.49-1.55) | 0.58 (0.20-1.63) |
| Tobacco smoking (any)^4^ | 0.74 (0.27-2.05) | 1.02 (0.18-5.72) |
| Anthropometry |  |  |
| BMI^5^ |  |  |
| <25 | 1.00 (ref) | 1.00 (ref) |
| 25-35 | 2.52 (1.30-4.87) | 2.55 (0.73-8.85) |
| >35 | 1.67 (0.54-5.14) | 0.59 (0.07-4.68) |
| Elevated waist circumference^5^ | 1.83 (0.98-3.41) | 1.88 (0.63-5.57) |

^1^99 missing

^2^105 missing

^3^17 missing

^4^22 missing

^5^18 missing

^6^75 missing
